# Germinated Thai Black Rice Extract Protects Experimental Diabetic Rats from Oxidative Stress and Other Diabetes-Related Consequences

**DOI:** 10.3390/ph10010003

**Published:** 2016-12-28

**Authors:** Chaiyavat Chaiyasut, Bhagavathi Sundaram Sivamaruthi, Noppawat Pengkumsri, Waranya Keapai, Periyanaina Kesika, Manee Saelee, Parichart Tojing, Sasithorn Sirilun, Khontaros Chaiyasut, Sartjin Peerajan, Narissara Lailerd

**Affiliations:** 1 Department of Pharmaceutical Sciences, Faculty of Pharmacy, Chiang Mai University, Chiang Mai 50200, Thailand; chaiyavat@gmail.com (C.C.); sivasgene@gmail.com or sivamaruthi.b@cmu.ac.th (B.S.S.); p_arkasus@hotmail.com (N.P.); p.kesika@gmail.com or kesika.p@cmu.ac.th (P.K.); manee_2533@hotmail.com (M.S.); ssirilun@gmail.com (S.S.); 2 Department of Physiology, Faculty of Medicine, Chiang Mai University, Chiang Mai 50200, Thailand; waranya_keapai@cmu.ac.th (W.K.); parichart_t@cmu.ac.th (P.T.); 3 Institute of Research and Development, Chiang Mai Rajabhat University, Chiang Mai 50300, Thailand; khonta_1@hotmail.com; 4 Health Innovation Institute, Chiang Mai 50200, Thailand; sartjin_p@yahoo.com

**Keywords:** diabetic mellitus, germinated black rice, γ-aminobutyric acid, anthocyanins, antioxidant, insulin

## Abstract

*Background*: Diabetes mellitus (DM), particularly type 2 DM (T2DM), is one of the most common metabolic disorder worldwide. The prevention measures and treatment strategies for DM are improving steadily. The current study explains the impact of germination on phytochemical content of Thai black rice (BR), and the influence of germinated BR extract (GBRE) supplementation on diabetic conditions in rats. *Methods*: BR was germinated and the phenolic, anthocyanin, and γ-aminobutyric acid (GABA) content of the extract were analyzed using HPLC and spectrophotometric methods. Streptozotocin-induced diabetic rats were supplemented with high and low doses of GBRE. The plasma glucose, insulin, cholesterol, triglyceride levels, antioxidant status, and antioxidant enzyme levels of treated animals were assessed using ELISA and spectrophotometric methods. *Results*: Germination enhanced the GABA content of BR, and GBRE intervention improved the total antioxidant capacity and antioxidant enzymes levels in diabetic rats. The plasma glucose, cholesterol, triglyceride levels, insulin resistance and glucose tolerance were reduced, and the degree of insulin secretion in rat plasma was significantly increased upon GBRE treatment. Both pre and post-treatment approaches showed the anti-diabetic ability of GBRE. In most of the analyzed parameters, GBRE was quite equal to the performance of drug-metformin. *Conclusions*: GBRE supplementation helps prevent and manage the consequences of DM.

## 1. Introduction

Diabetes mellitus (DM), especially Type-2 DM (T2DM), one the most common metabolic disorders worldwide, is due to faults in insulin secretion, or its action, or both. DM facilitates the development of serious diseases and disorders like retinopathy, neuropathy, nephropathy, heart attack, and stroke [1]. The T2DM incidence rates are increasing rapidly, and the universal incidence is projected to upsurge from 366 to 552 million people by 2030 [2,3]. DM is one of the obvious causes of death, morbidity, and significant socioeconomic problems, particularly in low and middle-income countries [3].

Genetic factors and lifestyle play a vital role in the instigation and development of T2DM, with diet being a key risk factor in T2DM initiation. It has already been proved that balanced diet management, regular exercise, and weight loss can prevent the onset of DM and also help to reduce the diabetic condition more effectively, along with medication [4,5]. A large portion of unprocessed whole grains in dietary patterns reduces the threat of T2DM. Several studies have proved that regular consumption of whole grain-based foods expands metabolic homeostasis and postpones the progress of T2DM and its consequences [6,7,8]. 

Oxidative stress and a mild level of inflammation can facilitate the development and pathogenesis of insulin resistance and diabetes complications. The antioxidants present in foods can prospectively help in reducing the risk of obesity-related diseases [9]. Oxidative stress is also associated with hyperglycemia, hyperlipidemia, and other diabetes-related consequences like cardiovascular complications, endothelial dysfunction and atherosclerosis [10,11,12,13]. The use of phytochemicals for the management and prevention of DM complications is promising [14].

Rice is one of the commonly consumed staple foods, which is a good source of antioxidants like γ-oryzanol, tocopherols, and tocotrienols. The contents of these phytochemicals differ among rice varieties, and are also influenced by the extraction methods [15,16]. The commonly used white rice has a higher glycemic index than that of colored rice and leads to high oxidative stress, which promotes the risk of T2DM. The total phenolic content of rice and rice bran are directly related to their in vitro antioxidant capability. Deng et al. reported the phenolic content and antioxidant properties of 24 cereal grains of China, and they found that the pigmented cereals (black, red, and purple rice) are rich in phenolic compounds and exhibited high free radical scavenging properties [17]. Zhang et al. demonstrated that the content of phytochemicals (phenolics, flavonoids, and anthocyanins) and antioxidant activity of 12 cultivars of black rice bran were significantly different [18]. Germination enhances the nutritional value of rice, primarily by increasing the content of γ-aminobutyric acid (GABA), which is a known neurotransmitter and regulator of blood pressure [19]. A few studies have reported the anti-diabetic activity of germinated brown rice [20,21].

Thai colored rice cultivars (black, brown, and red rice) are known for their high phenolic content and antioxidant properties. Pengkumsri et al. have reported that the black rice of northern Thailand is superior to brown and red rice, which was attributed to its phenolic content and free radical scavenging activity [15,16]. There are no detailed reports showing the anti-diabetic properties of germinated Thai black rice. Thus, the present study was performed with the aim of preparing germinated black rice extract (GBRE), analysis of its phytochemical content, and evaluation of the anti-diabetic properties of GBRE using a streptozotocin-induced diabetic rat model.

## 2. Materials and Methods

### 2.1. Sampling and Extraction

The black rice (Kum Payao), obtained from Payao Province, Thailand, was germinated in water (pH 7.0) at 40 °C for 24 h. The germination conditions were optimized in a separate study (unpublished data). The germinated black rice (GBR) was extracted with a solution of 4% acetic acid in 50% ethanol by shaking at 150 rpm at room temperature (RT) for 1 h, followed by centrifugation at 6000 rpm at RT for 20 min, and the supernatant was collected. The supernatant was then subjected to evaporation (50 °C) using an R-210 vacuum evaporator (Buchi Laboratoriums-Technik AG, Flawil, Switzerland) to acquire the concentrated extract. Then, the GBR extracts (GBREs) were stored at −20 °C until use. In the same way, non-germinated BR (NBR) was also extracted and served as a control for the determination of phytochemicals and GABA content.

### 2.2. Analysis of the BR Extract

#### 2.2.1. Determination of γ-Aminobutyric Acid (GABA) Content

The germinated black rice extract (GBRE) or non-germinated black rice extract (NBRE) were dissolved in 50% ethanol solution containing 4% acetic acid. The dissolved extract was filtrated using a 0.45 µm nylon filter. The sample was analyzed for GABA using gradient HPLC (LC 10AV, Shimadzu, Kyoto, Japan). A Shodex^®^ C18-4E (250 mm L × 4.6 mm ID, 5 µm) reversed phase column was used for separation with *o*-phthalaldehyde for post-column derivatization. The temperature of the column was set to 50 °C. Acetonitrile (A) and a buffer pH 3.5 (B) (0.0012% NaH_2_PO_4_, 0.0076% Na—*p*-toluenesulfinate in distilled water) was used as mobile phase. The following gradient system: 0–7 min (0% A), 7–9 min (60% A), 9–15 min (60% A), and 15–18 min (0% A) was used with a flow rate of 1 mL/min. The flow rate of the derivatization reagent was 0.5 mL/min. The fluorescence detector was set to 450/350 nm (emission/excitation) for the analysis [22].

#### 2.2.2. Phenolic Acids Determination

The total phenolic content of the GBRE and NBRE was determined by a modified Folin-Ciocalteu colorimetric method [15]. Briefly, 10× diluted Folin-Ciocalteu reagent (100 μL) was mixed with deionized water (1.5 mL), and extract (GBRE or NBRE, 200 μL) or gallic acid (positive control, 200 μL) at different concentrations. Saturated sodium carbonate (20%) was used to quench the reaction after 30 min of incubation at RT, and the absorbance was measured at 725 nm. Total phenolic content was represented as mg of gallic acid equivalent (mg GAE) per g of extract.

### 2.3. Anthocyanins Determination

The total anthocyanin content of the extract was determined as described earlier [15]. Briefly, extracts (GBRE or NBRE, 500 μL) or cyanidin chloride (positive control, 500 μL) at different concentrations were mixed with buffer solution pH 1.0 or 4.5 (2250 μL), incubated at RT for 20 min, and the absorbance measured at 510 and 700 nm. Total anthocyanin content was expressed as mg cyanidin chloride equivalent (mg CCE) per g of extract. The absorbance of positive control and sample (A) was calculated using Equation (1):

(1) $$A=[{\left(A_{510}\;-A_{700}\right)}_{\mathrm{pH}1.0}\;-{\left(A_{510}\;-A_{700}\right)}_{\mathrm{pH}4.5}$$

### 2.4. Animal Procedures

Adult male Wistar rats (180–200 g) were obtained from the National Laboratory Animal Center, Mahidol University (Bangkok, Thailand). The rats were maintained at 25 ± 2 °C with the dark-light cycle for 12 h as per the U.S. National Research Council guidelines. The animal experimental protocol was approved by the Ethical Committee for Using and Care of Animals, Faculty of Pharmacy, Chiang Mai University, Chiang Mai, Thailand (Certificate of ethical clearance no. 03/2015, dated 15 September 2015). A total of 72 rats were randomly separated into nine groups (eight rats per group) based on their initial body weight (BW, Table 1).

A single injection of 45 mg/kg BW. (i.p.) of streptozotocin (STZ) was used to induce a diabetic condition, and after ten days, rats with fasting plasma glucose levels of ≥250 mg/dL were classified as diabetic and used in this study [23]. Since the BR extract at up to 4000 mg/kg is considered safe for ICR mice (unpublished data), about 500 (low dose), and 1000 (high dose) mg/kg BW of GBRE was administrated to the respective animal groups along with normal diet (Commercial food no. C.P. 082, Perfect Companion Group Co., Ltd., Bangkok, Thailand). The low and high dose of GBRE was not supplemented to the experimental rats during the DM induction period (week 2 and 3) to monitor and confirm the diabetic condition without the interference of GBRE. The weight of experimental rats was carefully measured weekly during the period of the experiment. The fasting plasma glucose level was determined at every four weeks. After 12 weeks, animals were sacrificed and the blood samples were collected for the evaluation of antioxidant enzyme (catalase (CAT), superoxide dismutase (SOD), and glutathione peroxidase (GPx) levels, antioxidant capacity, lipid peroxidation inhibition ability, glucose level, triglyceride level, cholesterol level, and insulin level.

### 2.5. Total Antioxidant Capacity Determination

An 2,2′-azino-bis-3-ethylbenzthiazoline-6-sulphonic acid (ABTS) assay was performed to determine the total antioxidant capacity (TAC). Briefly, ABTS^•+^ working solution (2000 μL) was mixed with 100 μL of serum sample or Trolox (positive control), and incubated at RT for 3 min. The absorbance was measured at 734 nm. The results are expressed as mM Trolox equivalents antioxidant capacity (TEAC) of serum [15].

### 2.6. Lipid Peroxidation Inhibition Determination

Lipid peroxidation inhibition ability was assessed based on the concentration of malondialdehyde (MDA) formation [24]. In brief, serum (100 μL) and 1% sodium dodecyl sulfate (SDS, 100 μL) were mixed with 1% thiobarbituric acid (TBA, 200 μL). The reaction mixture was incubated at 95 °C for 30 min and rapidly cooled for 10 min. 50% trichloroacetic acid (100 μL) was used to terminate the reaction. The solution was mixed vigorously and centrifuged at 3000 *g* for 10 min. MDA product was measured at 540 nm by a Multimode Detector (DTX 880, Beckman Coulter Inc., Brea, CA, USA).

### 2.7. Antioxidant Enzymes Determination

The catalase (CAT), superoxide dismutase (SOD), and glutathione peroxidase (GPx) activities were measured in serum samples of the rat as reported by Yang et al. [25]. All experiments were carried out in triplicate, and the values were represented as U/mL.

### 2.8. Oral Glucose Tolerance Test

At the eleventh week of the treatment period, the oral glucose tolerance test (OGTT) was performed for each rat. All animals were starved overnight, and blood samples were collected to act as a baseline value (min-0). Then, a glucose solution (2 g/kg BW) was administrated by oral gavage feeding, and blood samples were collected from tail tip at 15, 30, 60 and 120 min after glucose supplementation and plasma glucose concentrations were measured using a commercial kit. The increase of plasma glucose concentrations following the glucose administration was expressed in terms of area under the curve (AUC) for glucose, using the trapezoidal rule [26].

### 2.9. Glucose, Triglyceride, and Cholesterol Determination

The plasma glucose, triglyceride, and total cholesterol concentrations were determined by the enzymatic colorimetric method using a commercial kit (Biotech, Bangkok, Thailand).

### 2.10. Insulin Determination

#### 2.10.1. Plasma Insulin Determination

The plasma insulin concentrations were measured by a Sandwich ELISA method (Rat/Mouse Insulin ELISA kit) as per the manufacturer’ instruction (LINCO Research Inc., St. Charles, MO, USA). 

#### 2.10.2. Homeostasis Model Assessment

The insulin resistance was assessed by the homeostasis model assessment of insulin resistance (HOMA-IR) [27]. HOMA-IR values are directly proportional to insulin resistance. i.e., the increase in HOMA-IR value indicates the increase of insulin resistance. HOMA-IR was calculated using Equation (2):

(2) $$\mathrm{HOMA}-\mathrm{IR}=\frac{\mathrm{fasting}\;\mathrm{insulin}\;\mathrm{level}\;\left(\frac{\mathrm{ng}}{\mathrm{mL}}\right)\;\times \;\mathrm{fasting}\;\mathrm{glucose}\;\mathrm{level}\;\left(\frac{\mathrm{mg}}{\mathrm{dL}}\right)}{405.1}$$

### 2.11. Statistics

The values were represented in mean ± SD. Analysis of variance (ANOVA) was used to test the differences among the values. Dunnett’s T3 *post hoc* test was used to determine the significant differences considering a level of significance at 95% confident intervals (*p* < 0.05) using the statistical SPSS software version 17 (SPSS Inc., Chicago, IL, USA). All the analytical experiments were performed in triplicate.

## 3. Results

### 3.1. Germination and Extraction

**Figure 1:**
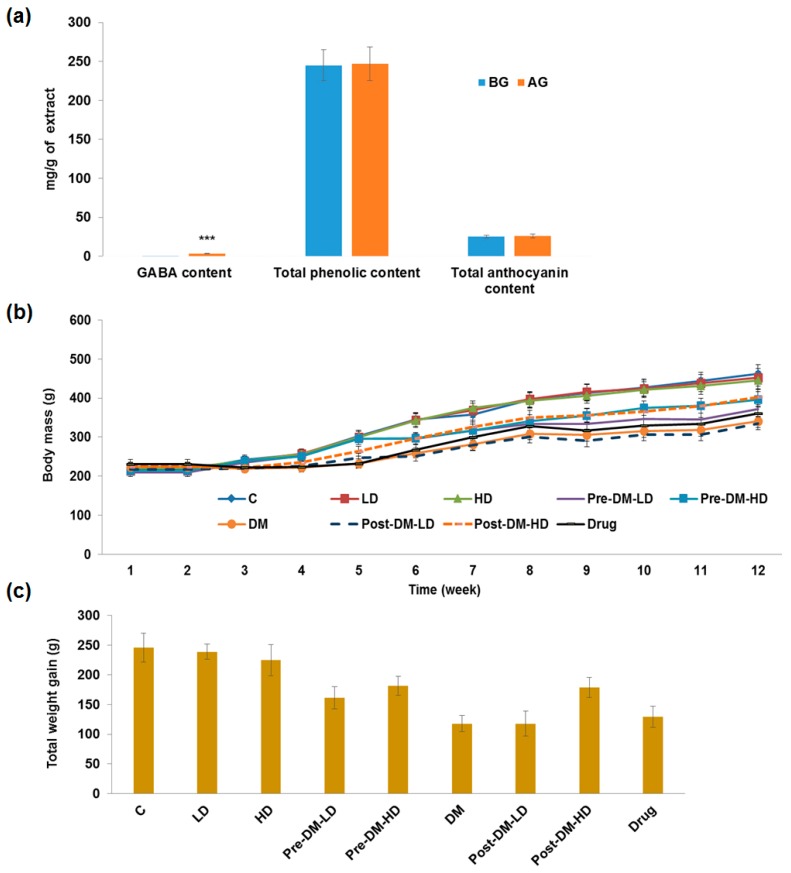
(**a**) The GABA, total phenolic, and anthocyanin content of germinated and non-germinated black rice. BG: Before germination, AG: After germination. Total phenolic content was represented as mg of gallic acid equivalent (mg GAE) per g of extract. Total anthocyanin content was represented as mg cyanidin chloride equivalent (mg CCE) per g of extract. *** Significant difference (*p* < 0.001); (**b**) Effect of GBRE supplements on the body mass of the experimental rats; (**c**) Total weight gain (total weight gained by the rat at end of the experiment (week 12) from the initial day of the experiment) in experimental rats. C: Control, LD: Low dose of GBRE to naïve rat, HD: High dose of GBRE to naïve rat, DM: Diabetes Mellitus induced rat, Pre-DM-LD: Low dose of GBRE to naïve rat and induced DM, Pre-DM-HD: High dose of GBRE to naïve rat and induced DM; Post-DM-LD: Low dose of GBRE to DM rat, Post-DM-HD: High dose of GBRE to DM rat.

The sampled black rice was germinated for 24 h under appropriate conditions and extracted as mentioned in the Materials and Methods section. The extracts were analyzed and the total phenolic content and total anthocyanin content of GBRE was 246 ± 21 mg GAE and 26 ± 2 mg CCE per g of GBRE, respectively. The GABA content of GBRE, was significantly (*p* < 0.001) higher when compared with the GABA content of NBRE (Figure 1a).

### 3.2. *In Vivo* Study

The anti-diabetic properties of the GBRE were evaluated in vivo using a STZ-induced diabetic rat model. Moreover, the present study examined pre- and post-diabetic conditions to explain the actual role of the test intervention, and with two different dosages. All the rats showed a gradual increase in the body mass during the experiment period (Figure 1b), while the DM group showed a more irregular total weight gain than that of the other groups (Figure 1c).

#### 3.2.1. Changes in Antioxidant Capacity

**Figure 2:**
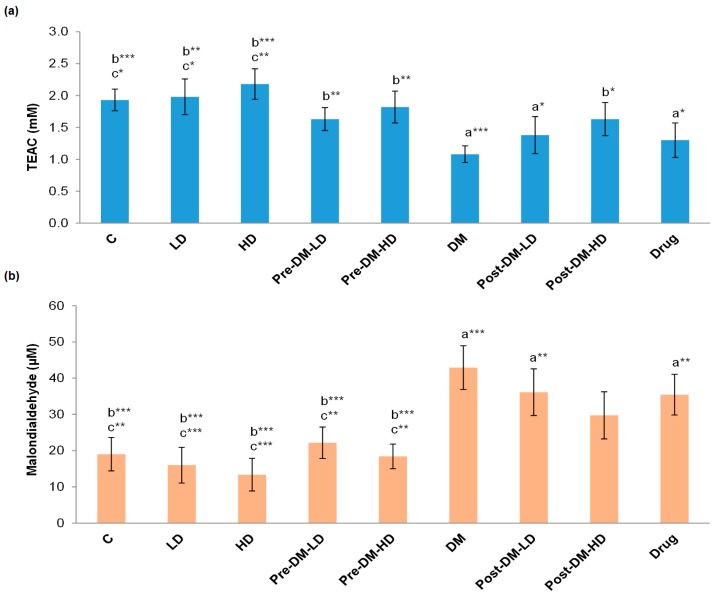
(**a**) Effect of GBRE supplement on the total antioxidant capacity of the experimental rats. The values are represented as Trolox equivalent of antioxidant capacity (TEAC); (**b**) MDA values of different rat groups. The MDA values are indirectly proportional to the lipid peroxidation inhibition ability. All the values are represented as mean ± SD of three determinations. C: Control, LD: Low dose of GBRE to naïve rat, HD: High dose of GBRE to naïve rat, DM: Diabetes Mellitus induced rat, Pre-DM-LD: Low dose of GBRE to naïve rat and induced DM, Pre-DM-HD: High dose of GBRE to naïve rat and induced DM; Post-DM-LD: Low dose of GBRE to DM rat, Post-DM-HD: High dose of GBRE to DM rat. The significant difference between the groups (a = when compared with control group; b = when compared with DM group; c = when compared with the drug group) were represented as * (*p* < 0.05), ** (*p* < 0.01), and *** (*p* < 0.001).

The antioxidant capacity was significantly (*p* < 0.001) reduced in the DM group when compared with the control group. LD, HD, and drug groups showed 1.98 ± 0.28, 2.18 ± 0.24, and 1.30 ± 0.27 mM of TEAC, whereas, the pre and post-treatment groups displayed better levels of antioxidant capacity pre-LD, pre-HD, post-LD, and post-HD, respectively (Figure 2a). The lipid peroxidation inhibition ability of rat serum was significantly (*p* < 0.001) decreased in the DM group when compared with the control group. LD, HD, and drug groups showed 15 ± 4, 13 ± 4, and 35 ± 5 µM MDA. The pre-treatment groups showed a significant (*p* < 0.001) elevation of lipid peroxidation inhibition ability and also post-treatment groups exhibited increased inhibition ability compared to the DM group (Figure 2b).

#### 3.2.2. Changes in Antioxidant Enzymes Level

**Figure 3:**
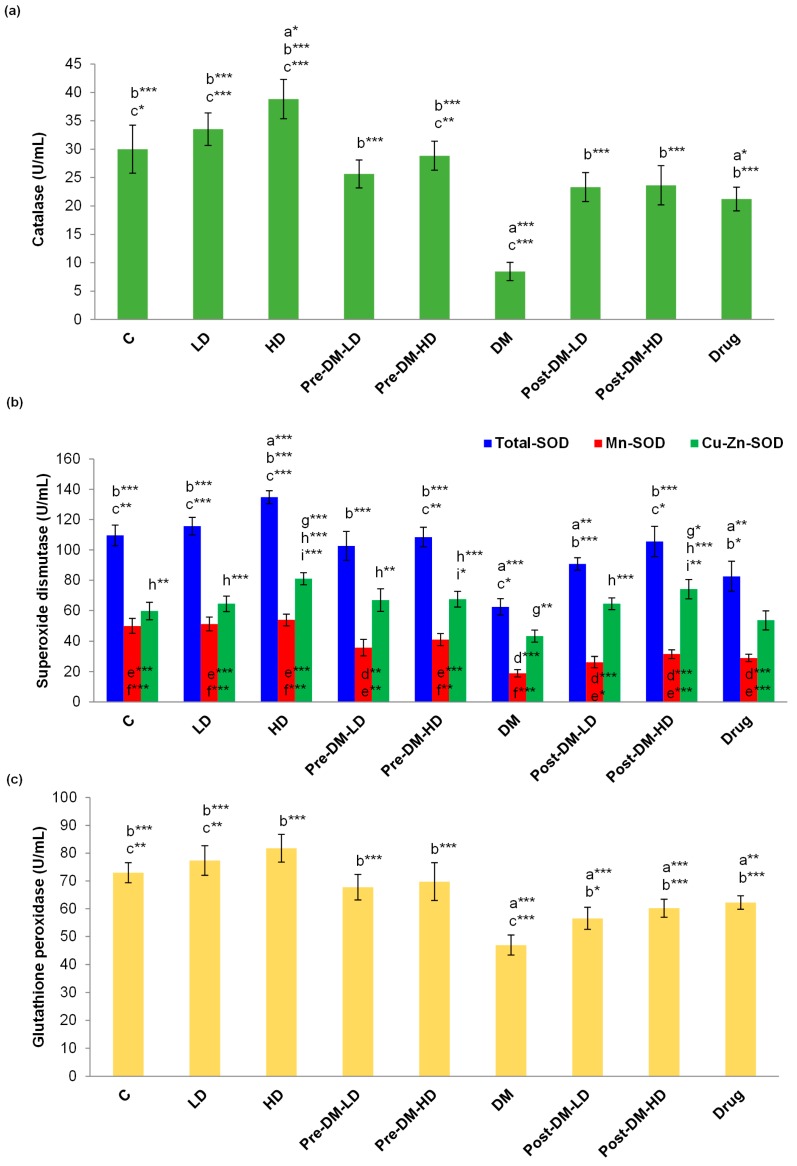
Effect of GBRE supplement on the antioxidant enzymes in the experimental rats. (**a**) Catalase; (**b**) Superoxide dismutase; (**c**) Glutathione peroxidase. C: Control, LD: Low dose of GBRE to naïve rat, HD: High dose of GBRE to naïve rat, DM: Diabetes mellitus-induced rat, Pre-DM-LD: Low dose of GBRE to naïve rat and induced DM, Pre-DM-HD: High dose of GBRE to naïve rat and induced DM; Post-DM-LD: Low dose of GBRE to DM rat, Post-DM-HD: High dose of GBRE to DM rat. The significant difference between the groups (a, d, g = when compared with control group; b, e, h = when compared with DM group; c, f, i = when compared with the drug group) were represented as * (*p* < 0.05), ** (*p* < 0.01), and *** (*p* < 0.001).

The representative antioxidant enzymes (catalase, superoxide dismutase (SOD), and glutathione peroxidase (GPx)) levels were studied in diabetic rat upon supplementation of GBRE. The level of catalase was significantly (*p* < 0.001) reduced in the DM group when compared with the control, LD, HD, and drug groups. While, pre-LD, pre-HD, post-LD, and post-HD groups showed relatively high catalase content (Figure 3a). The same scenario was observed for the GPx content and total SOD, Mn-SOD, and CuZn-SOD in experimental rats (Figure 3b,c).

#### 3.2.3. Profiles of Glucose, Glucose Tolerance, Triglyceride, and Cholesterol

**Figure 4:**
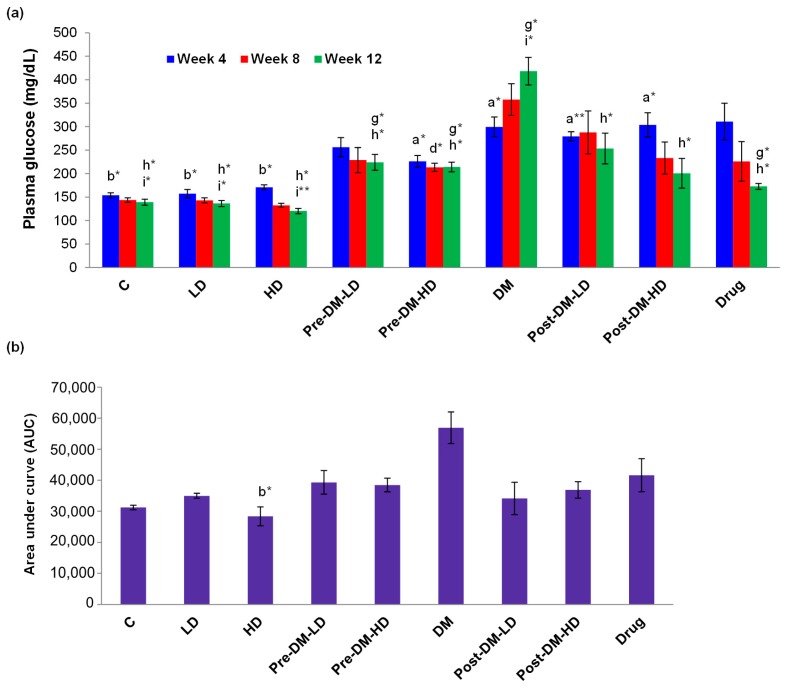
(**a**) Effect of GBRE supplement on the plasma glucose profile of rats during the experimental period. The plasma glucose level of the experimental rats was measured weekly (4, 8, and 12); (**b**) Effect of GBRE supplement on the glucose tolerance of experimental rats. The area under the curve (AUC) for glucose concentrations obtained from the oral glucose tolerance test were calculated using the trapezoidal rule [26]. C: Control, LD: Low dose of GBRE to naïve rat, HD: High dose of GBRE to naïve rat, DM: Diabetes Mellitus induced rat, Pre-DM-LD: Low dose of GBRE to naïve rat and induced DM, Pre-DM-HD: High dose of GBRE to naïve rat and induced DM; Post-DM-LD: Low dose of GBRE to DM rat, Post-DM-HD: High dose of GBRE to DM rat. The significant difference between the groups (a,d,g = when compared with control group; b,e,h = when compared with DM group; c,f,i = when compared with the drug group) were represented as * (*p* < 0.05), and ** (*p* < 0.01).

The fasting plasma glucose level of the rats was measured weekly (4, 8, and 12). The level of glucose was higher in DM-induced rats. The fasting plasma glucose level was reduced in the pre-treatment groups during the 8th week of the experimental period. A significant (*p* < 0.05) reduction in fasting plasma glucose was observed in post-treatment groups when compared with the DM group during the 12th week of the experimental period. The drug treated group also showed significant (*p* < 0.05) reduction in plasma glucose content when compared with the DM group during the 12th week of the experimental period (Figure 4a). The insulin sensitivity was determined by an oral glucose tolerance test (OGTT). The value of AUC for glucose in OGTT was reduced in both pre-, and post-treatment groups. The AUC for glucose in OGTT was less in the drug-treated group than that of the DM group, but higher than that of the experimental interventions either in pre- or post- treatment (Figure 4b).

**Figure 5:**
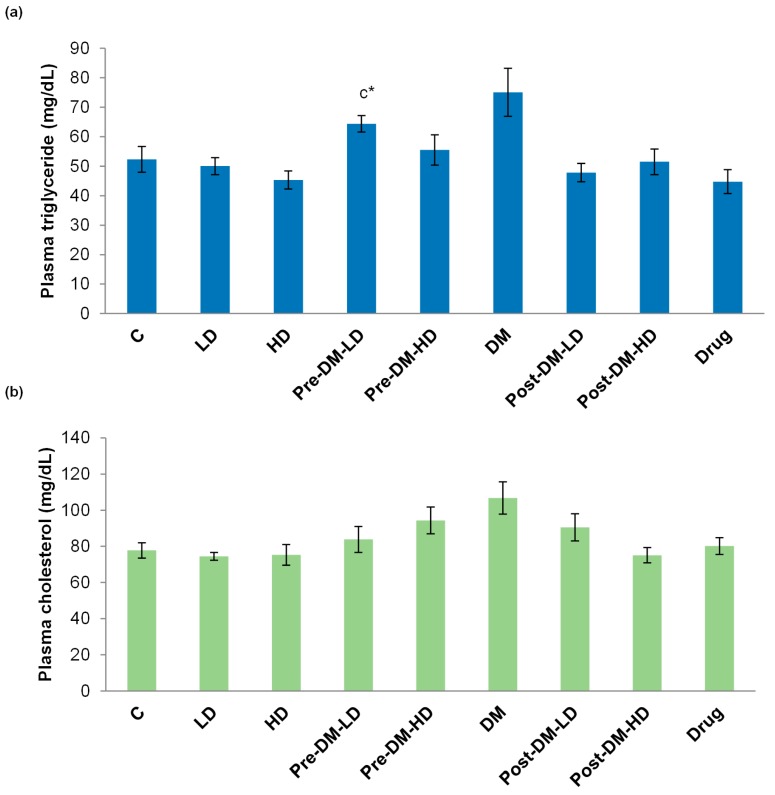
Effect of GBRE supplement on the (**a**) plasma triglyceride; and (**b**) cholesterol level in experimental rats. C: Control, LD: Low dose of GBRE to naïve rat, HD: High dose of GBRE to naïve rat, DM: Diabetes Mellitus induced rat, Pre-DM-LD: Low dose of GBRE to naïve rat and induced DM, Pre-DM-HD: High dose of GBRE to naïve rat and induced DM; Post-DM-LD: Low dose of GBRE to DM rat, Post-DM-HD: High dose of GBRE to DM rat. The significant difference between the groups (a = when compared with control group; b = when compared with DM group; c = when compared with the drug group) were represented as * (*p* < 0.05).

The triglyceride (TG) content of the plasma was decreased in the groups upon test interventions when compared with the DM group. The TG value of DM group was 75 ± 8 mg/dL, whereas the low dose of pre- and post-treatment showed only 64 ± 2, and 47 ± 3 mg/dL of TG, respectively. Likewise, the high dose of pre- and post-treatment showed only 55 ± 5, and 51 ± 4 mg/dL of TG, respectively. The drug treatment also reduced the TG content (Figure 5a). The total plasma cholesterol level was notably reduced by the test intervention in pre, and post treatments than that of the DM group. The drug treated group showed 80 ± 4 mg/dL of cholesterol in the plasma (Figure 5b).

#### 3.2.4. Insulin Profile and Insulin Resistance

**Figure 6:**
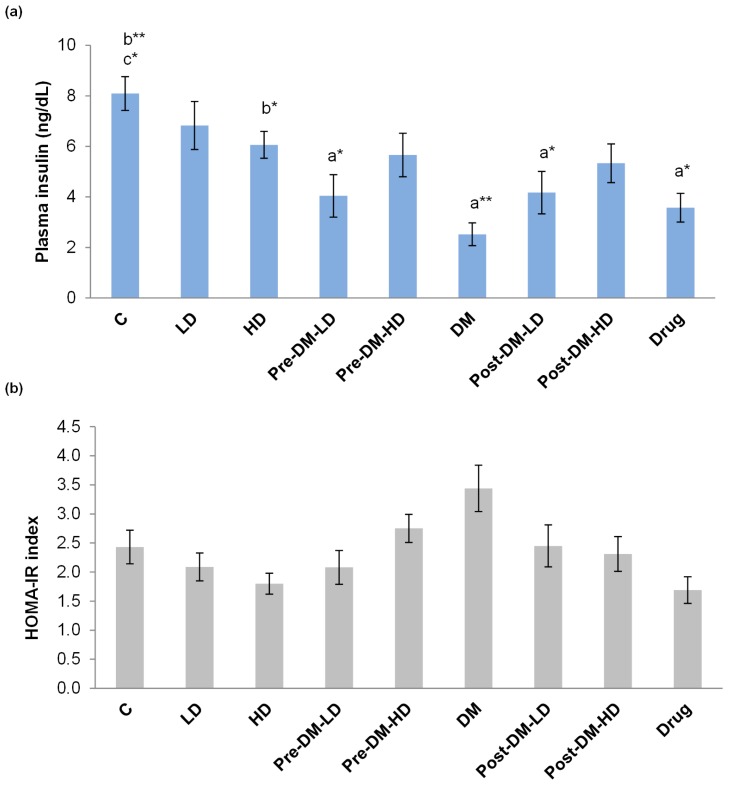
Effect of GBRE supplement on the insulin level (**a**) and insulin resistance; (**b**) in the experimental rats. C: Control, LD: Low dose of GBRE to naïve rat, HD: High dose of GBRE to naïve rat, DM: Diabetes Mellitus induced rat, Pre-DM-LD: Low dose of GBRE to naïve rat and induced DM, Pre-DM-HD: High dose of GBRE to naïve rat and induced DM; Post-DM-LD: Low dose of GBRE to DM rat, Post-DM-HD: High dose of GBRE to DM rat. The significant difference between groups (a = when compared with control group; b = when compared with DM group; c = when compared with the drug group) were represented as * (*p* < 0.05), and ** (*p* < 0.01).

The fasting plasma insulin level was improved by the test supplements in both pre-, and post-treatment groups when compared with the DM group. The DM group displayed only 2.52 ± 0.45 ng/dL of insulin, whereas the insulin level was increased in DM rats by the pre-treatment strategy. Likely, the post-treatment approaches in DM rat also improved the level of insulin. The improvement in insulin level by test supplement was relatively higher than that of the drug treatment group (Figure 6a). The insulin resistance of the rat was represented as HOMA-IR index. HOMA-IR index was reduced in treatment groups when compared with the DM group (Figure 6b).

## 4. Discussion

The present study has demonstrated the anti-diabetic property of GBRE using the STZ-induced diabetic rat model. The collected black rice samples were germinated, and then the GABA, total phenolic content, and total anthocyanin content of the extract were evaluated. The rice bran of the test BR was previously reported to exhibit a high content of phytochemicals and good antioxidant properties [15]. GABA content is increased in black rice during the germination process, and the bioactives, vitamins, minerals are related with the anti-diabetic capability of colored rice [20,28]. The GABA content in germinated rice of different varieties was reported by several researchers [21,29,30]. The recovery of GABA from germinated BR extract was observed to be higher in this study, which was a maximum recovery of GABA from BR. The germination process significantly (*p* < 0.001) increased the GABA content in BR (Figure 1a). It has been proved that GABA protects organs from oxidative stress, and is responsible for the hypocholesterolemic effect [31,32].

The germination process increased (4-fold) the phenolic content of the brown rice and the germinated brown rice exhibited high antioxidant activity [21]. In the present study, no significant changes were observed in phenolic and anthocyanin content among GBRE and NBRE (Figure 1a). The increased GABA content in GBRE possibly plays a significant role in the anti-diabetic properties of GBR, which was demonstrated in the current study.

The male Wistar rats were grouped and supplemented with test extract by pre-, and post-treatment approaches to investigate the role of GBRE in preventing and managing the diabetic condition. The body mass of the experimental rats of all the groups increased during the experiment (Figure 1b), whereas the difference in the total weight gain (from week 1 to week 12) varied between the control, DM, and treated groups (Figure 1c). Due to the diabetic condition, DM rats showed modest weight gains when compared with the control group, and our treatments improved the weight, especially for the high dose groups whose weight increased significantly more than that of others, regardless of the treatment strategy (Figure 1b,c). 

Imam et al. reported that the total antioxidant capacity (TAC) was increased from the baseline after the intervention of brown rice and germinated brown rice for 28 days [21]. The antioxidant capability of the rice depends on the phytochemical content, and BR is especially rich in phytochemicals (phenolic compounds, and anthocyanins). The tested GBRE was high in phenolic compounds and anthocyanins similar to the NBRE, while the GABA content of GBRE was significantly (*p* < 0.001) higher than that of the NBRE. Thus, the TAC was significantly improved in treatment groups compared to the DM group. The pre-treatment with a high dose of GBRE (1000 mg/kg BW.) prevented the reduction of TEAC levels in the rat (Pre-DM-HD), and these levels was equivalent to those of the naïve control rats. The post-treatment approaches also have enhanced the TAC of rat, but was not as effective as pre-treatment approaches. The drug treatment also apparently amended the TAC, whereby the test intervention was superior regarding the antioxidant capacity of experimental rats (Figure 2a). Lipid peroxidation inhibition ability was enhanced among the pre-treated groups similar to that of the naïve level, especially in the pre-DM-HD group. Post-treatments and drug supplementations did not improve the lipid peroxidation inhibition ability in diabetic rats (Figure 2b).

Tocopherols, flavonoids, carotenoids, and ascorbic acid are the core antioxidants for the treatment of T2DM [33,34]. Rice phytochemicals, rich in tocopherols, reduce oxidative stress by enhancing catalase, superoxide dismutase, and other antioxidant enzyme activity both in vitro and in vivo [35]. Black rice extract intervention amended serum lipid profile and boosted the fatty acid metabolism [36]. In the current study, the antioxidant enzymes (catalase, SOD, and GPx) level was improved in the GBRE supplemented rats when compared with the antioxidant enzyme levels in the DM group. Catalase level was increased in pre- and post-treated groups compared to the drug-treated group. The pre-treatment procedure was more effective in improving the level of catalase and GPx in diabetic rat than the post-treatment approach. A rise in the concentration of total-SOD, Mn-SOD, and CuZn-SOD was observed in the diabetic rats due to the test intervention when compared with the DM group (Figure 3). Posuwan et al. reported the anti-diabetic ability of colored rice that exhibited the reduction of oxidative stress [37]. The results of the present study also clearly revealed that GBRE supplementation improves the levels of antioxidant enzymes, and TAC of the host system, thereby reducing the consequences of diabetes mellitus.

Several studies have explained the reduction of plasma glucose by rice phytochemicals and germinated rice [38,39]. In the present study, the plasma glucose level was gradually reduced in treated groups and increased in the DM group. Pre-treatment reduced the fasting plasma glucose levels. High dose of GBRE more successfully decreased the fasting plasma glucose level in both the pre- and post-treatment procedures. Overall, the GBRE suppresses the glucose level in plasma after four weeks of intervention, which was comparable with the metformin group (Figure 4a). 

Chronic hyperglycemia is an indicator of T2DM, which can be described by determining the glucose tolerance. The pre-germinated brown rice slightly reduces the plasma adipocytokines, HbA1c level, and insulin resistance in the rat, by which it prevents the development of T2DM [19]. In the present study, glucose tolerance level was reduced (reduction in AUC) among GBRE supplemented rats (Figure 4b). The glucose tolerance was not influenced by the dose of the extract in the pre-treatment groups, whereas, the post-treatment groups exhibited dose-dependent activity. The untreated DM group presented a constant increase in AUC value, which was the indication of magnification of the diabetic condition. The tested GBRE extract was effective when compared with the drug (metformin) group regarding glucose tolerance (Figure 4b).

About seven weeks of black rice diet reduced the plasma triglyceride (TG) level in rats more than that of the control rats [38]. The rice-based herbal porridge reduced the cholesterol level in the rat [39]. Kima et al. reported that the consumption (for 12 weeks) of brown rice lees extract reduced the waist circumference of fifteen T2DM patients. The test group (15 patients who consumed the brown rice lees extract) exhibited a significant increase in aspartate transaminase and alanine transaminase when compared with the control group (15 patients who consumed mixed grains), whereas serum glucose, OGTT, insulin level, and HOMA index of the test group was not significant when compared with the control group, while the control group showed reduced plasma cholesterol level [40]. In the present study, the plasma TG, and cholesterol levels were effectively reduced by GBRE intervention in the diabetic rat (Figure 5). The post-treatment (Post-DM-HD) strategy was effective regarding the cholesterol content (Figure 5b). The treatment approaches were effective in maintaining the TG levels, except for the pre-DM-LD group. The GBRE was as effective as drug treatment concerning TG levels (Figure 5a). 

Treatments (pre- and post-) successfully reduced the insulin resistance and increased the insulin level. GBRE supplementation was superior to metformin regarding insulin level (Figure 6). The results clearly indicate the anti-diabetic ability of GBRE in diabetic rats. Several studies have proved that anthocyanin affects glucose absorption by hindering the discharge of glucose during digestion, insulin secretion, and action, and lipid metabolism [41,42]. It is known that black rice is one of the more nourishing dietary sources of anthocyanins, and easily degradable upon physical treatments [43]. 

Recent studies have reported screening and characterization of lactic acid bacteria, which further aid to develop GABA-rich functional foods in the form of fermented plant beverages and probiotic drinks [22,44,45,46], but germinated colored rice represent a straightforward and delicious source of GABA, and no extensive processing is required to obtain the same. The anti-diabetic properties are attributed to the elevated GABA content and the unaffected levels of anthocyanins and phenolic content of GBRE.

Regulation of glucose release and transport is one of the ways by which a functional diet reduces the consequences of T2DM. Thus, the establishment of functional foods for global consumption has been recommended to prevent and/or manage T2DM [47]. The current study explained the anti-diabetic capability of germinated Thai black rice variety in the STZ-induced diabetic rat model. The GBRE exhibited tangible anti-diabetic properties in both pre- and post-treatment procedures. In some parameters, the pre-treatment approach was better than that of the post-treatment group, and vice versa. In most of the analyzed parameters, the performance of GBRE was quite equal to that of the drug metformin.

## 5. Conclusions

The abundance of phenolic compounds, anthocyanin content, and an increase in GABA concentration in germinated Thai black rice was demonstrated. The impact of GBRE supplementation in a DM-induced rat model was explained and it was found that GBRE improved the antioxidant status of diabetic rats and diminished the magnitude of DM. Finally, the results revealed that germinated black rice supplementation helps prevent and manage the consequences of DM. Germinated black rice supplemented with other dietary combinations may thus provide an effective method for the prevention and treatment of DM.

## Figures and Tables

**Table 1:** The grouping of experimental rats, the dosage of GBRE supplementation and study period.

No	Group (G)	Week 1	Week 2, 3	Week 4–12
1	C (G1)	ND & NDW	ND & NDW	ND & NDW
2	LD (G2)	500 mg/kg BW. of GBRE, ND & NDW	Buffer injection (i.p.) *, ND & NDW	500 mg/kg BW. of GBRE, ND & NDW
3	HD (G3)	1000 mg/kg BW. of GBRE, ND & NDW	Buffer injection (i.p.) *, ND & NDW	1000 mg/kg BW. of GBRE, ND & NDW
4	PreLD (G4)	500 mg/kg BW. of GBRE, ND & NDW	STZ injection (i.p.) *, ND & NDW	500 mg/kg BW. of GBRE, ND & NDW
5	PreHD (G5)	1000 mg/kg BW. of GBRE, ND & NDW	STZ injection (i.p.) *, ND & NDW	1000 mg/kg BW. of GBRE, ND & NDW
6	DM (G6)	ND & NDW	STZ injection (i.p.) *, ND & NDW	ND & NDW
7	PostLD (G7)	ND & NDW	STZ injection (i.p.) *, ND & NDW	500 mg/kg BW. of GBRE, ND & NDW
8	PostHD (G8)	ND & NDW	STZ injection (i.p.) *, ND & NDW	1000 mg/kg BW. of GBRE, ND & NDW
9	Drug (G9)	ND & NDW	STZ injection (i.p.) *, ND & NDW	50 mg/kg BW. of MFN, ND & NDW

Note: C: control, LD: low dose, HD: high dose, PreLD: pre-treatment with low dose of GBRE, PreHD: pre-treatment with high dose of GBRE, PostLD: post-treatment with lose dose of GBRE, PostHD: post-treatment with high dose of GBRE, Drug: metformin, ND: normal diet, NDW: normal drinking water, DM: Diabetes Mellitus, GBRE: germinated black rice extract, i.p.: intraperitoneal injection, STZ: streptozotocin (45 mg/kg BW.), MFN: metformin (50 mg/kg BW.), *: The initial day of week 2 GBRE and the drug were supplemented to rat along with normal diet.
